# The role of gene expression and symbiosis in reef-building coral acquired heat tolerance

**DOI:** 10.1038/s41467-022-32217-z

**Published:** 2022-08-03

**Authors:** Marie E. Strader, Kate M. Quigley

**Affiliations:** 1 Department of Biological Sciences, Auburn University, Auburn, AL USA; 2 Minderoo Foundation, Perth, WA Australia

**Keywords:** Evolution, Climate-change ecology

## Abstract

Predicting how reef-building corals will respond to accelerating ocean warming caused by climate change requires knowledge of how acclimation and symbiosis modulate heat tolerance in coral early life-history stages. We assayed transcriptional responses to heat in larvae and juveniles of 11 reproductive crosses of *Acropora tenuis* colonies along the Great Barrier Reef. Larvae produced from the warmest reef had the highest heat tolerance, although gene expression responses to heat were largely conserved by cross identity. Juvenile transcriptional responses were driven strongly by symbiosis – when in symbiosis with heat-evolved Symbiodiniaceae, hosts displayed intermediate expression between its progenitor *Cladocopium* and the more stress tolerant *Durusdinium*, indicating the acquisition of tolerance is a conserved evolutionary process in symbionts. Heat-evolved Symbiodiniaceae facilitated juvenile survival under heat stress, although host transcriptional responses to heat were positively correlated among those hosting different genera of Symbiodiniaceae. These findings reveal the relative contribution of parental environmental history as well as symbiosis establishment in coral molecular responses to heat in early life-history stages.

## Abstract (web summary)

Coral responses to ocean warming depend not only on the coral themselves, but also on their symbionts. Here, the authors experimentally assess how symbiosis and gene expression plasticity contribute to the heat stress responses of a common coral.

## Introduction

Ocean temperatures are rising beyond the physiological limits of many marine taxa, driving rapid responses of organisms to counteract extinction either through range shifts, acclimation, or adaptation. Coral reefs globally are experiencing more frequent and extreme heat stress anomalies, which can induce short-term mortality and diminish recovery times, resulting in extreme reductions in population sizes. These changes drive community shifts to alternative stable states devoid of hard, reef-building corals and limit suitable substrate for coral recruitment, creating a negative feedback loop that further threatens coral reefs^1,2^. Despite global population declines, thermally tolerant genotypes exist^3,4^, which may facilitate the rapid evolution of heat tolerance for applied conservation practices like assisted evolution^5^. Genetic and environmental factors influence the adaptive and acclimation potential of organisms in response to global environmental change. The relative roles of these factors depend on the environment experienced currently or in previous generations through either past selection pressures or acclimation^6^. In corals with large population sizes spanning extensive ranges across temperature gradients, as with some species of *Acropora* in the Indo-Pacific, the potential for acclimation and adaptation to higher temperatures indicative of some future warming scenarios likely exists^7,8^. However, the full picture of how each of these processes contributes to the future persistence of coral populations is yet to be fully understood, especially when the compounding effects of symbiosis on host physiology and responses to thermal stress in early life-history stages are taken into account.

Genetic interventions to increase coral thermal tolerance are being considered worldwide to allow time for climate change action and mitigation. These methods include facilitating rapid acclimation, modifying symbiont communities (eukaryotic and prokaryotic), selectively breeding corals, and experimentally evolving their symbionts^5^. The utilization of assisted gene flow methods, a form of selective breeding, across temperature gradients on the Great Barrier Reef (GBR) can increase heat tolerance in corals^3,9^ without severe trade-offs in growth, survival, and symbiont acquisition^10^. Furthermore, when multiple approaches are combined, there is a synergistically positive increase in heat tolerance^9^ resulting from increased adaptive genetic diversity in regions associated with heat tolerance^4^. In this study, we use several assisted evolution methods synergistically to tease apart the role that gene expression plasticity (acclimation), and microbial community manipulation (symbiosis) have on the acquired heat tolerance of early life-history stages of a wide-spread, habitat-building, Indo-Pacific coral species. Colonies of *Acropora tenuis* were selectively bred from five reef sites stretching over 6° of latitude on the GBR, resulting in 11 distinct crosses, including purebred and hybrid gametic crosses (Supplemental Fig. 1 but see^11^ for full information on collection, coral spawning, reproduction, and cross design). Of these sites, three were sourced from the far North GBR (Curd (CU), Long Sandy (LS), and Sandbank 7 (SB)), with Curd Reef experiencing the highest mean sea surface temperature (SST) and two sites from the central GBR (Davies Reef (DR) and Backnumbers (BK)) (Supplemental Fig. 1). To quantify acclimation effects contributing to early life-history survival under heat stress, we characterized gene expression of both larvae and juveniles assayed under ambient conditions, heat stress, and under different symbiotic states. Transcriptional responses are a key determinant to survival and recovery during and after heat stress^12^ and corals employ multiple transcriptional strategies in response to environmental change^13–16^. We identify key genes associated with high larval survival under heat stress, which was specific to crosses produced from dams originating from Curd Reef, but report an overall conserved transcriptional response to heat across all crosses. Alternatively, host transcriptional responses to heat are functionally different in juveniles and are strongly associated with the symbiotic community. Juvenile symbiosis with a heat-evolved *Cladocopium* strain has host transcriptional profiles similar to juveniles hosting *Durusdinium*, and contributed to improved juvenile survival under heat stress. These results have important implications for understanding the molecular mechanisms behind heat tolerance acquisition and for the manipulation of symbiont communities and selective breeding in coral restoration.

## Results

### Larvae display conserved gene expression response to heat stress

**Fig. 1:**
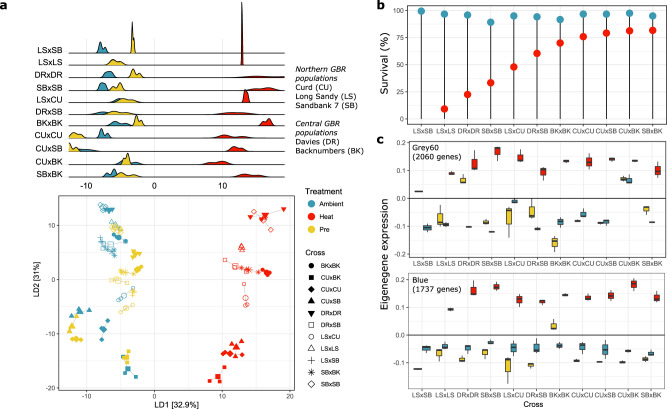
Larval gene expression responses to heat stress in genetically distinct crosses. **a** Discriminant analysis of principal components (DAPC) with heat treatment symbolized by color and cross ID symbolized by shape. The first linear discriminant axis separates the heat treatments, 27 °C for “pre” (*n* = 33 pools of 10 larvae each) and “post ambient” (*n* = 33 pools of 10 larvae each), 35.5 °C for “post heat” (*n* = 30 pools of 10 larvae each), with density plots showing the distribution of samples in each cross and treatment along LD1, ranked from low to high survival in heat stress. **b** Larval survival in heat and ambient treatments. **c** Eigengene expression for two WGCNA modules positively, and significantly correlated with larval response to heat. For each cross, the origin of dams is denoted first, and the origin of sires is second (*n* = 3 pools of 10 larvae each per cross per treatment). Boxplots include the median values (center lines), upper and lower quartiles (box limits), 1.5× interquartile range (whiskers), and outliers (points).

Larval gene expression (GE) was quantified to assess if plastic responses in gene expression to heat stress varied depending on site of origin or parental identity. Larval survival under heat stress varied between crosses, with larvae produced from dams sourced from far Northern GBR sites exhibiting higher thermal tolerance (Fig. 1b). The cross with the lowest thermal tolerance (LSxSB) did not have any larvae survive the heat treatment (Fig. 1b, Supplementary Fig. 2). GE was examined in aposymbiotic larvae experiencing ambient conditions prior to the heat treatment (“pre”), larvae after exposure to simulated heat stress (35.5 °C for 56 hours, “post heat”), and a simultaneous control temperature of 27 °C (“post ambient”). Therefore, the “pre” larval treatment provided transcriptomic baselines of GE between genetic crosses while “post heat” and “post ambient” comparisons show a baseline for cross-specific heat responses without the confounding effect of symbiosis found in the post-metamorphic phase. Using a principal coordinates analysis (PCoA), we find that GE patterns in larvae were driven by treatment (“pre”, “post ambient”, “post heat”), explaining 29.2% of the variation in survival (*p*_*adonis*_ < 0.001, Fig. 1a, Supplemental Fig. 2). Discriminant function of principal components (DAPC), a superior method compared to PCoA for partitioning the variance between populations^17^, recapitulates PCoA patterns showing both treatment and cross-specific effects on larval GE. Using DAPC, we observe that “pre” treatment GE often lies midway between both of the post-treatment GE responses along DAPC-1 (Fig. 1a). Weighted gene co-expression network analysis^18^ identified groups of genes (the grey60 module, 2069 genes, and the blue module, 1737 genes), which were positively correlated with larval heat treatment (*R*^2^ = 0.84, *p* = 3e-26, and *R*^2^ = 0.94, *p* = 2e-44, respectively.) (Fig. 1c, Supplementary Fig. 3), but did not strongly correlate with larval survival under heat stress, which varied by cross (Fig. 1, Supplemental Fig. 3). This indicates a relatively conserved gene expression response to heat between crosses, despite differences in larval survival. The blue and grey60 modules also showed negative and positive correlations, respectively, with survival under ambient conditions (Supplementary Fig. 3). Gene Ontology (GO) categories enriched in genes within the blue module include “regulation of response to stress” and “nf-kappaB signaling”, while the grey60 module has GO enrichment of “protein folding” and “response to endoplasmic reticulum stress”, categories which are also enriched under a range of environmental stress responses^16^ and implicated in the selective breeding for heat tolerance^4^, suggesting their critical importance in a heritable response to heat tolerance acquisition (Supplementary Data 10).

This cross-site breeding design allowed us to further clarify the impact of the local reef, and thus the environmental history, on the molecular responses to thermal stress in coral offspring. Cross identity explained 29.9% of the variation in larval GE (Supplemental Fig. 2, *p*_adonis_ < 0.001), suggesting host genetics plays a vital role in larval heat tolerance. Larvae produced from maternal colonies from the warmest site, Curd Reef, which also exhibited high survival to heat stress (CUxBK, survival 81.25% ± 4.2^11^, Fig. 1b), demonstrated unique larval GE responses under elevated temperature. In the CUxBK cross, we observed an upregulation of thermal response genes (genes in the grey60 module) under “pre” and “post ambient” conditions, indicative of genetic assimilation (Fig. 1c)^19^, a type of response that has been described in heat-tolerant corals sourced from shallow lagoons in other reef locations^20^. A cross with the paternal contribution from Curd (LSxCU) exhibited intermediate GE responses to heat that were between crosses with Curd dams and the other crosses produced from cooler reefs (Fig. 1a), further indicating that individual colonies from Curd reef may be differentially adapted to heat stress given their northern, inshore location. Specifically, the expression of 11 genes differentiated larvae with Curd dams in their responses to heat, including an nf-Kappa-B inhibitor (Supplementary Fig. 4). Additionally, modeling in DESeq2 of the “pre heat” larval samples with survival under subsequent heat stress identified 438 genes predictive of larval survival in *A. tenuis* (Supplementary Data 8), which are further referenced throughout as “thermal tolerance” genes. GO categories “cell cycle processes” and “ribosome” were significantly enriched in genes that were predictive of larval survival (Supplementary Data 5). The association of these genes in crosses produced from parents of Curd origin suggests that these genes may play a role in the local adaptation to the more extreme, inshore, warmer conditions experienced by this reef.

### Juvenile gene expression varies based on symbiont community

**Fig. 2:**
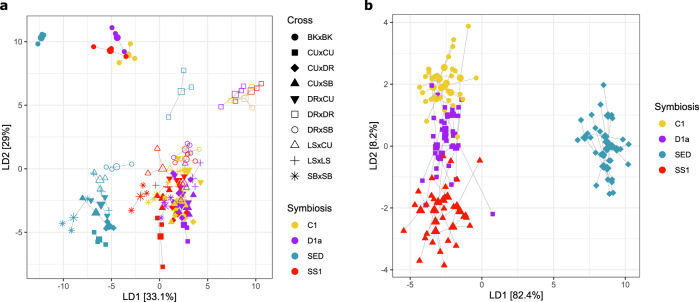
Juvenile gene expression responses to symbiosis. Discriminant analysis of principal components (DAPC) on gene expression of juveniles hosting different symbioses under ambient conditions. DAPC was performed on variance stabilized data (VSD) grouped by cross+symbiosis (**a**) and with the effect of cross removed by *limma* (**b**). For each cross, origin of dams is denoted first, and the origin of sires is second.

**Fig. 3:**
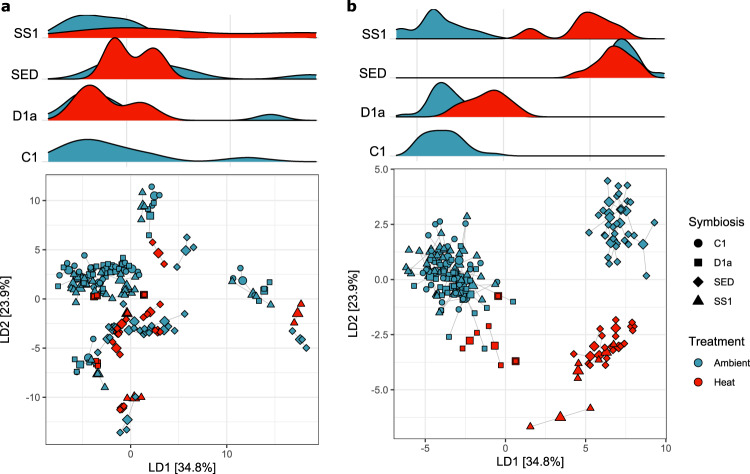
Juvenile gene expression responses to symbiosis and heat stress. Discriminant analysis of principal components (DAPC) on gene expression of juveniles hosting different symbioses under ambient and heat conditions. DAPC was performed on variance stabilized data (VSD) grouped by cross+treatment+symbiosis (**a**) and with the effect of the cross removed by *limma* (**b**) with density plots showing the distribution of samples in each symbiosis and treatment along LD1.

**Fig. 4:**
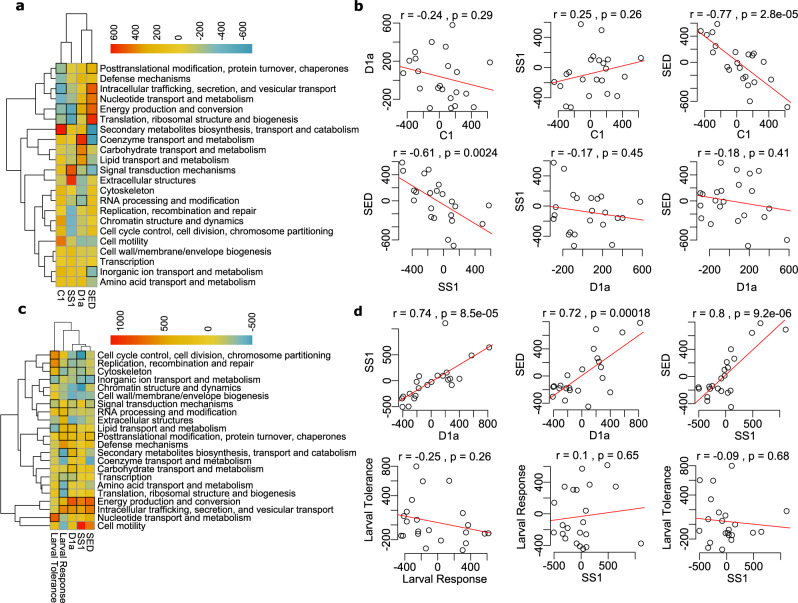
Functional similarities in juvenile responses to hosting different symbionts and exposed to heat stress. Hierarchical clustering analysis of KOG enrichments in juveniles hosting different symbioses at ambient conditions (**a**) and host responses to heat in juveniles hosting different symbioses (**c**). Correlations of KOG delta rank values for each comparison under ambient conditions (**b**) and symbiosis responses to heat (**d**). KOG categories in bolded squares denote the significance of Mann–Whitney *U* test at *p*_*adj*_ < 0.05. Exact Benjamini-Hochberg adjusted *p* values for each comparison are in Supplementary Data 9. Color scale in **a** and **c** are KOG delta rank values.

While mechanisms elucidating acquired heat tolerance have been examined in aposymbiotic larvae^3,4^ (Fig. 1, Supplementary Figs. 2–4), no studies have yet resolved GE responses to heat or variable symbiotic states in juvenile corals. Arguably, quantifying the role of population origin, plasticity, and symbiosis in this critical life-history stage is urgently needed as juvenile survival and sensitivity to environmental stress are key for the conservation and restoration of coral populations globally. To address this, larvae from the aforementioned crosses were induced to metamorphose and then subsequently exposed to a range of cultured or wild Symbiodiniaceae treatments. Juveniles were infected with cultured *Cladocopium goreaui* (“C1”), *Durusdinium trenchii* (“D1a”), a heat-evolved species of *Cladocopium goreaui* (“SS1”)^21^, or a diverse community of putatively heat-tolerant symbionts from the sediments collected from Curd (“SED”), with the dominant infection of each symbiont type confirmed with transcripts mapping to each genus of Symbiodiniaceae (Supplementary Fig. 6). Juvenile *Acropora* uptake diverse communities of Symbiodiniaceae from their environment, a dynamic process comprised of a taxonomically variable symbiont consortium that plays a substantial role in heat tolerance acquisition and survival in juveniles^10,22^. At ambient temperatures, juvenile host GE is mainly clustered by symbiont identity although a significant effect of the cross was also observed (Fig. 2). A permutational multivariate ANOVA revealed that the cross explained 15.7% of the variance in juvenile GE under ambient conditions with symbiont treatment explaining 10.6%. In partitioning the variance using DAPC, we find that two crosses (DRxDR and BKxBK) showed unique GE clustering (Fig. 2) and despite close proximity of these two sites on the offshore GBR, appear to have unique histories of selection and acclimation. Juveniles in the “SED” treatment hosted a highly diverse symbiont community, comprised of >300 taxonomic groups^11^, which may explain the pattern of high GE divergence in these juveniles compared to those hosting cultured symbionts. This emphasizes the influence of symbiont diversity in the modulation of host physiology, even during early ontogeny (Fig. 3, Supplementary Fig. 6). Moreover, juveniles in the ambient “SED” treatment exhibited the downregulation of genes associated with signal transduction, coenzyme transport, and secondary metabolite synthesis, and upregulation of energy production and conversion, and intracellular trafficking, secretion, and vesicular transport (Fig. 4a, Supplementary Data 9), compared to juveniles with cultured symbionts. These functional categories are a hallmark of host regulation of the symbiotic state^23^, likely in the midst of the winnowing process^24,25^, and are most likely representative of juvenile host gene expression in situ on the reef. In juveniles, gene modulation in response to different symbiotic states included changes in genes associated with cell receptor signaling, cellular adhesion proteins, transcription, cellular stress responses, immunity, and the cell cycle (Fig. 4a, Supplementary Data 6). Juveniles hosting the SS1 strain, which was experimentally evolved to tolerate higher temperatures, upregulated genes associated with biological adhesion molecules, and regulation of cell morphogenesis (Supplementary Data 6). These functional categories of genes have been previously implicated as signatures of selection and convergence in the evolution of vertical transmission in corals^26^, suggesting that these pathways are specifically modulated by the host when the symbioses are perturbed.

### Larvae and juveniles have different responses to heat stress

Under heat stress, juveniles hosting C1 experienced substantial mortality and are thus not represented in this study, while juveniles hosting SS1, experimentally evolved from C1, exhibited higher survival under heat stress. Survival of juveniles hosting SS1, D1a, and communities from the sediments all exhibited similarly high survival under heat stress^11^ as well as significantly positively correlated functional responses to heat (Fig. 4d). While juveniles hosting SS1 at ambient conditions hosted a majority of *Cladocopium* transcripts, SS1 juveniles under heat stress all exhibited elevated proportions of *Durusdinium*-derived transcripts (Supplementary Fig 6). Therefore, the heat stress itself may have promoted a shift towards *Durusdinium*-derived transcripts in SS1 infected juveniles, potentially facilitating their thermal tolerance. Further, juveniles hosting SS1 showed positively significant molecular responses to heat that are indicative of corals hosting D1a as seen in the KOG correlations between these two responses (*R*^2^ = 0.74 *p* = 8.5e-05, Fig. 4d), with stronger correlations to those hosting communities from the sediments (*R*^2^ = 0.8 *p* = 9.2e-06, Fig. 4d). Despite these overall functional similarities, key differences were observed that highlight the unique interactions between symbiont identity, symbiont community, and coral host origin that contribute to juvenile heat stress responses. For example, under heat stress, SS1 juveniles significantly downregulated cell cycle control, cell division, and chromosome partitioning genes compared to the other two symbioses (Fig. 4d), indicative of a trade-off between growth and thermal tolerance. Compared to the other symbioses, D1a juveniles downregulated signal transduction genes (Fig. 4d), genes likely to be involved in symbiosis establishment and maintenance.

Heat-stress responses in juveniles hosting all symbiont treatments exhibited non-significant correlations with the heat stress responses in larvae (Fig. 4d). This underscores that heat tolerance is likely regulated by different molecular mechanisms in larvae and juvenile *Acropora tenuis*. Juveniles in all symbiont treatments showed strong upregulation of energy production and conversion genes compared to the larval heat stress response (Fig. 4c). Larval heat tolerance also showed no functional correlations with either larval or juvenile responses to stress, suggesting different molecular mechanisms confer tolerance and response in early life-history stages.

## Discussion

Heat tolerance may be regulated via several divergent evolutionary pathways, including genetic fixed effects or via changes in gene expression^7,27,28^. These mechanisms are likely linked, where the interplay between genetic fixed effects and gene expression can be broadly connected by mechanisms like expression QTLs (reviewed in ref. 27). Here we use a unique experimental design to quantify the synergistic effects of acquired heat tolerance via gene expression plasticity and symbiosis in early life-history stages. We profiled gene expression in both aposymbiotic larval and symbiotic juvenile stages at pre-stress, ambient, and heat-stress treatments across 11 distinct genetic crosses between and amongst northern and central GBR reef sites of *Acropora tenuis*. We found that selective breeding between disparate sites revealed relatively conserved gene expression profiles underlying key differences in larval and juvenile heat tolerance between crosses, although mechanisms of heat tolerance differed substantially between these disparate life-history stages. Juveniles hosting SS1 under ambient conditions exhibited an intermediate heat response situated between D1a and their progenitor taxa C1 (Supplementary Fig. 5). These results suggest that the evolution of heat tolerance in the SS1 strain likely follows a conserved, or constrained, evolutionary pattern. In Symbiodiniaceae, given the recapitulation of molecular host responses to SS1 and D1a, but “residual” or ancestral similarity of SS1 to C1, it is likely that further experimental heat selection may be needed to completely convert the SS1 capacity for heat tolerance that is in line with the canonically stress-tolerant D1a. Together these data quantify, simultaneously, the relative contribution of gene expression and symbiosis in critical early life-history stages and provide an applied foundation for optimizing genetics-based methods for the enhancement of coral survival.

## Methods

### Coral spawning, larval and juvenile experiments

Reproductive *Acropora tenuis* coral colonies were collected across 6° of latitude and were spawned at the National Sea Simulator at the Australian Institute of Marine Science (AIMS) to produce purebred and hybrid crosses. The full details of reef locations (Supplemental Fig. 1), coral colony collections, spawning, and reproductive crossing can be found in^11,29^. Larvae produced from these crosses were sampled for RNAseq (“pre” samples) prior to exposure at 0 hrs (directly before the heat ramp started) and cohorts of those larval cultures were subjected to control and heat stress for 36 hours (27 and 35.5 °C) in replicates of *n* = 6 (“post” exposure samples). Separate cohorts of each larval cross not used in heat trials were then induced to settle using sterilized crustose coralline algae as a settlement cue, exposed to the four symbiont treatments for symbiosis infection, and then exposed to control and heat stress conditions for 56 days (27 °C and 32 °C). The three cultured symbionts were grown under LED, providing illumination set at 60 m^−2^ s^−1^ photosynthetically active radiation measured with a Li-Cor light sensor for a 14:10 light:dark photoperiod. Before inoculation, cells were measured using a Neubauer Haemocytometer (*n* = 3 replicates/symbiont) to make up a final cell density of 1 × 10^5^ ml^−1^.

### RNA extraction and tag-seq

All larvae and juveniles were preserved in *RNAlater* and stored at −20 °C until library preparation. Larvae and juveniles were both preserved at the ambient and heat treatments once the heat treatment reached ~50% mortality. Ten pooled larvae and two juveniles were extracted per cross/treatment due to low RNA concentrations per larva and juvenile. Extractions were performed using an RNAqueous kit (Ambion) and homogenized with an additional bead-beating step in 100 µl Lysis solution with ~6 glass beads (MPBio lysing matrix C) on the FastPrep (5.5 m/s for 30 sec × 2 times). Total RNA quantity was normalized to 375 ng for larval samples and 30 ng for juvenile samples using an RNA Pico Chip kit on the Agilent 2100 Bioanalyzer. Due to the high mortality of juveniles infected with C1 in the heat treatment, there was not adequate RNA to sequence these individuals. Samples were then selected and normalized based on RNA concentration and RIN (15 ng/µl for larvae, mean RIN = 6.6 and 2.5 ng/µl for juveniles, mean RIN = 6.9) and sent to Genomic Sequencing and Analysis Facility at the University of Texas at Austin for custom library preparation and SR50 tag-based sequencing on the Illumina Hi-Seq 2500.

### Bioinformatics and statistical analysis

Read processing of RNAseq reads employed established wrapper scripts for tag-seq (https://github.com/z0on/tag-based_RNAseq)^30^. In summary, reads were trimmed of adaptor sequences, PCR duplicates were removed and filtered for quality using cutadapt (v.2.10)^31^. For the larval dataset, sequencing yielded an average of 4,138,973 raw reads per sample (min: 351,509, max: 5,369,230, median: 4,133,164), which was reduced to a mean of 2,643,781 reads per sample after trimming (min: 247,979, max: 3,332,923, median: 2,686,320). For the juvenile dataset, sequencing yielded an average of 3,648,218 raw reads per sample (min: 1,688,621, max: 5,973,561, median: 3,619,747.5), which was reduced to an average of 1,695,570 reads per sample after trimming (min: 705,818, max: 3,285,162, median: 1,673,007.5) (Supplementary Data 1 and 2). Although recent work in corals using similar approaches has yielded deeper read depths (>35 M; see reference 32), the tag-seq approach used here only sequences the 3’ end of the transcripts, resulting in substantially fewer reads required to assess gene expression, as the full assembly of full-length transcripts is not needed^33^. To verify the appropriateness of mapping references, sequences were mapped to both the conspecific (*A. tenuis*^34^) and heterospecific (*A. millepora*^35^) references. Overall, the results were similar, but the *A. tenuis* genome was selected based on the higher mapping efficiency (Supplementary Data 1 and 2). Cleaned reads were mapped to transcripts mined from the *Acropora tenuis* genome using Bowtie2 (v.2.2.9)^36^. The mapping efficiency of larval and juvenile samples averaged 63.4% and 54.4%, respectively (Supplementary Data 1 and 2). Gene counts were compiled using a custom perl script (samcounts.pl). Gene expression was analyzed using DESeq2 (v.1.30.1), implemented in R (v.4.0.4)^37^. Only highly abundant genes with >10 mean counts across samples were retained for statistical analysis: 11,192 genes for the larval data set, 9311 genes for the juvenile dataset. For the larval dataset, differentially expressed genes (DEGs) were identified using a model that defined fixed factors of treatment (“pre”, “post ambient”, “post heat”) and cross. Juvenile gene expression was analyzed in two different ways. First, only samples under ambient conditions were considered and a model was run specifying fixed effects of symbiosis and cross. Second, all samples including juveniles exposed to heat were modeled with fixed effects of treatment, symbiosis, and cross. Significant DEGs were defined by a false discovery rate adjusted *p* value of <0.05 (Supplementary Data 3 and 4). Stat values from each comparison were used for GO enrichment analysis using the GO_MWU package (v.1.2)^38^. This test involves a two-sided Mann–Whitney *U* test to identify functional enrichment of GO terms without having to set arbitrary cut-offs.

Variance stabilized counts data from each model were output and used for additional multivariate analyses. Variance partitioning was determined using a principal coordinates analysis (PCoA) of Manhattan distances and the significance of each fixed effect was assessed using the adonis function in R. Additionally, the variance associated with each fixed effect was analyzed using a DAPC using the package adegenet (v.2.1.4)^39^.

Weighted gene co-expression network analysis (WGCNA^18^, v.1.69), implemented in R (v.3.6.1), was used to further identify groups, or modules, of genes whose expression significantly correlates with known factors in the larval dataset. A signed network was constructed using a soft threshold power of 18, a minimum module size of 30, and a module merging threshold of 10% dissimilarity. Module trait relationships are calculated using the cor() function in R, utilizing a Pearson correlation. GO enrichment analysis on gene modules was performed using Fisher’s exact test for genes present within the module or not using the GOMWU framework^38^. For the juvenile dataset, a KOG enrichment analysis was performed. This analysis bins genes into 23 broad functional groups, reducing the complexity of functional annotations. The KOG_MWU function calculates delta rank values for these 23 broad functional groups using gene-level log2-fold changes generated from pairwise comparisons between treatments in DESeq2^40^. For juveniles under ambient conditions, comparisons were between juveniles with a specific symbiont type (C1, D1a, SS1, or SED) against all other samples at ambient (Fig. 2b). For the comprehensive juvenile dataset, heat responses were examined using pairwise comparisons between ambient and heat for each symbiont type (C1 ambient vs. C1 heat, for example). As all the SS1 heat samples contained noticeable proportions of *Durusdinium* transcripts, a comparison was performed 1) retaining all six of these samples and 2) removing those samples with approximately half of the transcripts derived from *Durusdinium*. Both comparisons yielded similar results (Supplementary Fig. 7). Additional comparisons were included in this analysis, including larval thermal tolerance (“pre” samples modeled against survival under heat stress) and larval thermal response (“post ambient” vs. “post heat”).

### Reporting summary

Further information on research design is available in the Nature Research Reporting Summary linked to this article.

## Supplementary information

Supplementary Information

Supplementary Data 1

Supplementary Data 2

Supplementary Data 3

Supplementary Data 4

Supplementary Data 5

Supplementary Data 6

Supplementary Data 7

Supplementary Data 8

Supplementary Data 9

Supplementary Data 10

Reporting Summary

## Data Availability

ITS2 sequences are available at NCBI SRA at PRJNA720058 and RNAseq files are accessible through the NCBI GEO repository at GSE176051. Publicly available data used for our analysis include the *Acropora tenuis* genome: http://aten.reefgenomics.org/; and the *Acropora millepora* genome: https://www.ncbi.nlm.nih.gov/bioproject/767661.

## Code availability

Analysis scripts are located at: https://github.com/LaserKate/AGF18_RNAseq under Zenodo 10.5281/zenodo.6820007^41^ (https://zenodo.org/badge/latestdoi/355383542).
