# Supplementary material for: The role of gene expression and symbiosis in reef-building coral acquired heat tolerance
Source: Nat Commun. 2022 Aug 3;13:4513. doi: 10.1038/s41467-022-32217-z (PMC9349291; doi:10.1038/s41467-022-32217-z)
Supplement: Supplementary file 1 — Supplementary Information [file 41467_2022_32217_MOESM1_ESM.docx]

**The role of gene expression and symbiosis in reef-building coral acquired heat tolerance**

Marie E. Strader and Kate M. Quigley

Supplementary Information

Figures


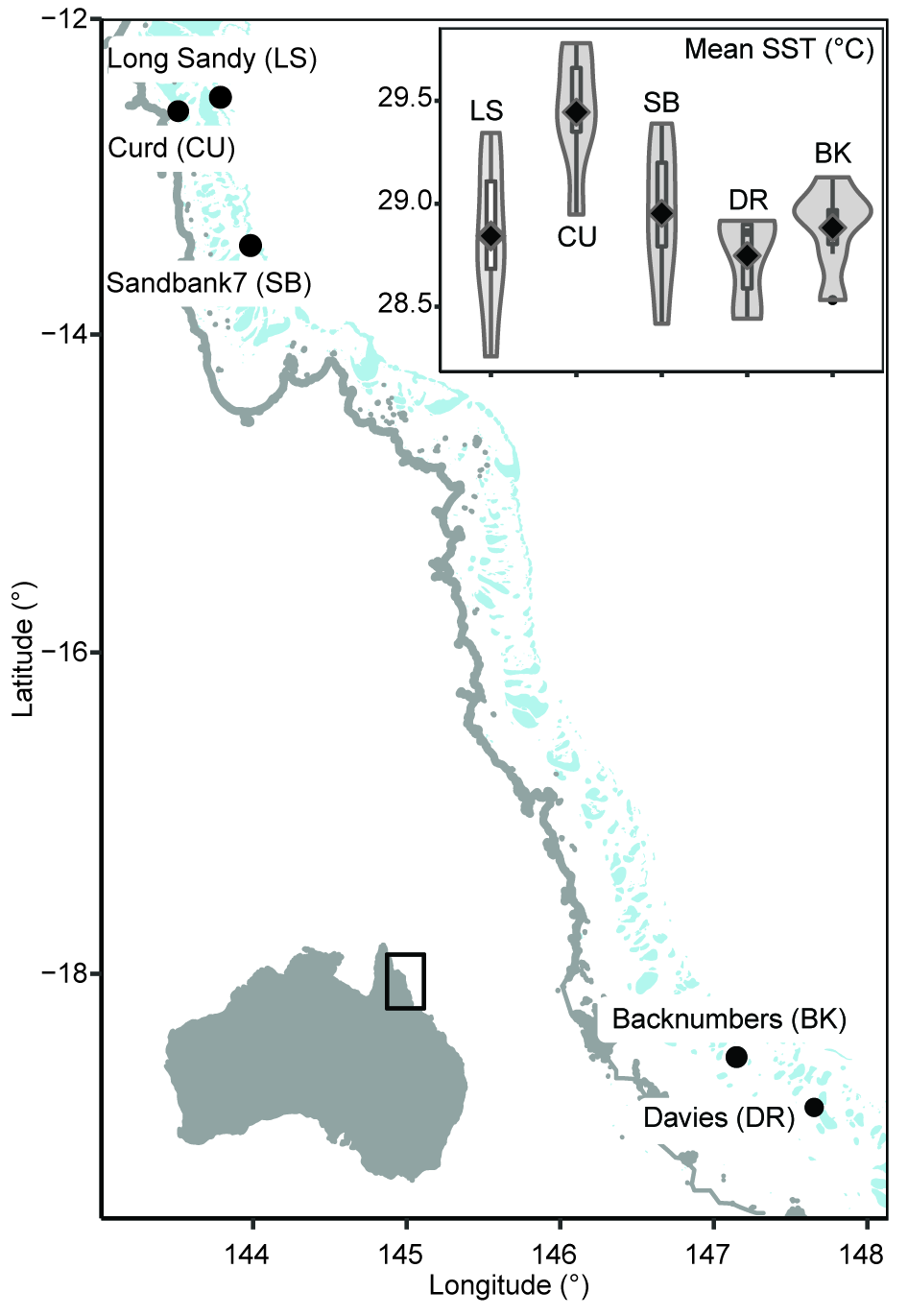


**Supplementary Figure 1.** Site locations along the northern and central Great Barrier Reef were gravid *Acropora tenuis* colonies were collected for spawning and subsequent larval and juvenile experiments. Inset: mean annual temperatures averaged across daily recordings for a full year (01/01/2013 – 28/02/2018). Data is freely available for downloaded from eReefs (1 km grid-cell resolution). Three populations are from the far North GBR (Curd (CU), Long Sandy (LS) and Sandbank7 (SB)), with Curd reef experiencing the highest mean sea surface temperature (inset), and two populations are from the central GBR (Davies (DR) and Backnumbers (BK)).

**Supplementary Figure 2**: Principal Coordinates Analysis of larval samples using variance stabilized gene expression counts data with mean counts >10 across all samples. Crosses are derived from three populations from the far North GBR (Curd (CU), Long Sandy (LS) and Sandbank7 (SB)), and two populations are from the central GBR (Davies Reef (DR) and Backnumbers (BK)). For each cross, origin of dams is denoted first, and origins of sires is second. Treatment explains 29.2% of the variation in GE while cross explains 29.9% of the variation in GE (PERMANOVA, *p*_adonis_<0.001.).

**Supplementary Figure 3:** WGCNA module trait relationships for larval samples. Genes with >10 mean counts across larval samples were input into WGCNA analysis. A signed network was constructed using a soft threshold power of 18, a minimum module size of 30 and module merging threshold of 10% dissimilarity. Gene modules (denoted by colors, for example “ME blue”) were correlated to quantitative and categorical traits. Quantitative traits include “survivalheat” and “survivalambient” and remaining traits are categorical, with “1” and “0” as the presence or absence of a sample in that category. Within each square are the Pearson correlation and the associated Fisher test p-value, in parentheses. “N” refers to a north population (Curd (CU), Long Sandy (LS) and Sandbank7 (SB), and “C” refers to central GBR populations (Davies (DR) and Backnumbers (BK)).

**Supplementary Figure 4:** Gene expression of genes that are significantly responsive to heat (“post heat”; n=30 pools of 10 larvae each) compared with ambient (“post ambient”; n=33 pools of 10 larvae each) in crosses with Curd reef dams (*p.adj*=<0.05 for CUBK heat vs. ambient; *p.adj*=<0.05 for CUCU heat vs. ambient; and *p.adj*=<0.05 for CUSB heat vs. ambient) but not in any of the other larval crosses (*p.adj*=>0.05). “Pre” samples included in the plots for reference (n=33 pools of 10 larvae each). Red symbols and lines denote crosses that have Curd reef dams (T); blue symbols denote crosses that do not have Curd reef dams (F). Error bars signify standard error of variance stabilized count data across all samples within a factor. NA is a gene with no annotation.

**Supplementary Figure 5:** Principal Coordinates Analysis of juveniles hosting different symbiosis (58 days post settlement) under ambient conditions only (a) and in ambient (blue) and heat (red) (b). Under ambient conditions, cross explains 15.9% of the variation in GE while symbiosis explains 10.5% of the variation in GE (*p*_adonis_<0.001). Under ambient and heat conditions (b), cross explains 14.5% of the variation, heat treatment explains 5.37% of the variation and symbiosis explains 7.9% of the variation (PERMANOVA, *p*_adonis_<0.001).

**Supplementary Figure 6:** Proportion of RNAseq reads mapping to the respective transcriptomes of four genera of Symbiodiniaceae for each treatment (C1=juveniles infected with cultured *Cladocopium goreaui*, D1a=juveniles infected with *Durusdinium trenchii* culture, SED=juveniles allowed to uptake a natural symbiont community from Curd reef sediments, SS1=juveniles infected with a culture of a heat-evolved species of *Cladocopium goreaui*). Stars (*) indicate juveniles infected with the SS1 strain that experienced the heat treatment. Fractions correspond to transcripts in each of the four clades/genera (fracA= *Symbiodinium,* fracB= *Breviolum*, fracC= *Cladocopium*, fracD= *Durusdinium*). ﻿*Symbiodinium* and *Breviolum* transcriptomes are from Bayer et al. (2012), and *Cladocopium* and *Durusdinium* transcriptomes are from Ladner, Barshis, and Palumbi (2012). Concatenated transcriptome file acquired from Manzello et al. (2019).

**Supplementary Figure 7:** KOG correlations amongst juvenile samples with varying Symbiodiniaceae treatments (C1=juveniles infected with cultured *Cladocopium goreaui*, D1a=juveniles infected with *Durusdinium trenchii* culture, SED=juveniles allowed to uptake a natural symbiont community from Curd reef sediments, SS1=juveniles infected with a culture of a heat-evolved species of *Cladocopium goreaui*). Five (sample IDs 30, 57, 94, 125, 157) out of the six SS1 heat treatment samples had approximately half of the symbiont reads mapping to *Durusdinium* (Supplementary Figure 6). The remaining SS1 heat sample (sample ID 93) also had noticeable levels of *Durusdinium*. We did a comparison where the data was analysed 1) retaining all the SS1 heat samples (six total samples), top row, and 2) removing the five samples with approximately half the reads mapping to *Durusdinium*, bottom row. Since the overall results are similar retaining the samples, and it gives stronger power for the analysis, all six samples were retained.
