# Supplementary material for: The role of gene expression and symbiosis in reef-building coral acquired heat tolerance
Source: Nat Commun. 2022 Aug 3;13:4513. doi: 10.1038/s41467-022-32217-z (PMC9349291; doi:10.1038/s41467-022-32217-z)
Supplement: Supplementary file 12 — Reporting Summary [file 41467_2022_32217_MOESM12_ESM.pdf]

nature portfolio

Corresponding author(s): Marie Strader, Kate Quigley

Last updated by author(s): July 12, 2022

Reporting Summary

Nature Portfolio wishes to improve the reproducibility of the work that we publish. This form provides structure for consistency and transparency in reporting. For further information on Nature Portfolio policies, see our Editorial Policies and the Editorial Policy Checklist.

Statistics

For all statistical analyses, confirm that the following items are present in the figure legend, table legend, main text, or Methods section.

n/a	Confirmed	
☐	☒	The exact sample size (*n*) for each experimental group/condition, given as a discrete number and unit of measurement
☐	☒	A statement on whether measurements were taken from distinct samples or whether the same sample was measured repeatedly
☐	☒	The statistical test(s) used AND whether they are one- or two-sided<br>*Only common tests should be described solely by name; describe more complex techniques in the Methods section.*
☐	☒	A description of all covariates tested
☐	☒	A description of any assumptions or corrections, such as tests of normality and adjustment for multiple comparisons
☐	☒	A full description of the statistical parameters including central tendency (e.g. means) or other basic estimates (e.g. regression coefficient) AND variation (e.g. standard deviation) or associated estimates of uncertainty (e.g. confidence intervals)
☐	☒	For null hypothesis testing, the test statistic (e.g. *F*, *t*, *r*) with confidence intervals, effect sizes, degrees of freedom and *P* value noted<br>*Give P values as exact values whenever suitable.*
☒	☐	For Bayesian analysis, information on the choice of priors and Markov chain Monte Carlo settings
☒	☐	For hierarchical and complex designs, identification of the appropriate level for tests and full reporting of outcomes
☐	☒	Estimates of effect sizes (e.g. Cohen's *d*, Pearson's *r*), indicating how they were calculated

Our web collection on statistics for biologists contains articles on many of the points above.

Software and code

Policy information about availability of computer code

Data collection	ITS2 sequences are available at NCBI SRA at PRJNA720058 and RNAseq files are accessible through the NCBI GEO repository at GSE176051.<br>Publicly available data used for our analysis include the Acropora tenuis genome: http://aten.reefgenomics.org/; and the Acropora millepora genome: https://www.ncbi.nlm.nih.gov/bioproject/767661<br>No software was used for data collection.
Data analysis	Analysis scripts are located at: https://github.com/LaserKate/AGF18_RNAseq under Zenodo DOI 10.5281/zenodo.6820007 (https://zenodo.org/badge/latestdoi/355383542).<br><br>Software used to analyse RNAseq data is specified in the document. The following software was used:<br>established wrapper scripts for tag-seq (https://github.com/z0on/tag-based_RNAseq)<br>DESeq2 (v.1.30.1), implemented in R (v.4.0.4)<br>Stat values from each comparison were used for GO enrichment analysis using the GO_MWU package (v.1.2), implemented in R (v.4.0.4)<br>discriminant analysis of principal components (DAPC) using the package adegenet (v.2.1.4), implemented in R (v.4.0.4)<br>Weighted gene co-expression network analysis (v.1.69), implemented in R (v.3.6.1).

For manuscripts utilizing custom algorithms or software that are central to the research but not yet described in published literature, software must be made available to editors and reviewers. We strongly encourage code deposition in a community repository (e.g. GitHub). See the Nature Portfolio guidelines for submitting code & software for further information.

Data

Policy information about availability of data

All manuscripts must include a data availability statement. This statement should provide the following information, where applicable:

- Accession codes, unique identifiers, or web links for publicly available datasets
- A description of any restrictions on data availability
- For clinical datasets or third party data, please ensure that the statement adheres to our policy

ITS2 sequences are available at NCBI SRA at PRJNA720058 and RNAseq files are accessible through the NCBI GEO repository at GSE176051.
Publically available data used for our analysis include the Acropora tenuis genome: http://aten.reefgenomics.org/; and the Acropora millepora genome: https://www.ncbi.nlm.nih.gov/bioproject/767661 under Zenodo DOI 10.5281/zenodo.6820007 (https://zenodo.org/badge/latestdoi/355383542).

The GEO repository has been made publicly available as of July 8, 2022

Human research participants

Policy information about studies involving human research participants and Sex and Gender in Research.

Reporting on sex and gender	N/A
Population characteristics	N/A
Recruitment	N/A
Ethics oversight	N/A

Note that full information on the approval of the study protocol must also be provided in the manuscript.

Field-specific reporting

Please select the one below that is the best fit for your research. If you are not sure, read the appropriate sections before making your selection.

☐ Life sciences ☐ Behavioural & social sciences ☒ Ecological, evolutionary & environmental sciences

For a reference copy of the document with all sections, see nature.com/documents/nr-reporting-summary-flat.pdf

Ecological, evolutionary & environmental sciences study design

All studies must disclose on these points even when the disclosure is negative.

Study description

Derived statistics presented are defined as independent observations of n= independent larval or juvenile survival based on the number of remaining individuals per replicate well at the final sampling time point.

Experimental metadata of detailed replicates for larval treatments are found on the github repository in file J19188meta.csv. For "pre", and "post-ambient" there were 33 larval replicates. For "post-hot" there were 30 replicates. Each replicate represented 10 pooled larvae. Each of the 11 crosses was replicated 3 times within each of those 3 treatment groups. There was a total of 96 larval samples.

Experimental metadata for juvenile data is found on the github repository in file J19234meta.csv. Of the juvenile samples, 119 were in ambient conditions with 27 in the heat treatment. Each of the 10 crosses was represented in the juvenile dataset 12-18 times. The symbiont treatments had 29 samples in C1, 38 samples in D1, 43 samples in SED, and 36 in SS1. There was a total of 146 juvenile samples.

Wild coral colonies were collected throughout the Great Barrier Reef and reproductively crossed at a land based facility. Larvae were reared and subjected to a heat stress Larvae were then induced to settle, then exposed to symbionts and then exposed to heat stress. Both larvae (10 pooled) and juveniles (2 pooled) were sampled for gene expression and assayed using RNAseq. The experimental design was hierarchical.
Larval replicates were sampled for RNAseq for each of the crosses prior to the heat stress at time 0 and 56 hours at 27°C and 35.5°C treatments. For survival measurements, individual larvae were counted within net-wells in replicate plates within each temperature treatment. Each larval survival measurement represents a discrete sample measurement. The unit of measure is the number of individual replicate wells containing larvae.
Juvenile replicates RNAseq samples were taken in each of the crosses after 58 days at 27 and 32°C. Survival measurements represent individual juvenile survival. Each represents a discrete sample measurement. The unit of measure is the number of individual replicate juveniles per replicate well, per replicate plate, per replicate tank for each temperature and symbiont treatment.

Research sample	A research sample involved individual genotypes of corals from different life-stages. For example, a survival measurement of a single larvae or a single juvenile coral. Phenotypes were measured directly on the individual basis. Individual samples were collected as it provided greater power to the experimental design compared to bulk measurements. For gene expression samples, each larvae sample was a pool of 10 larvae where juveniles were pools of 2 individuals. The species used was Acropora tenuis. Adults were collected from across the northern and central Great Barrier Reef (coordinates provided), of at least 3 to 5 years of age, wild-caught. Both larvae and juveniles were reproductive crossed (manipulated) in a quantitative genetic cross design. This species was chosen given its amenability to coral reproductive biology and settlement cues, necessary for the experiment.
Sampling strategy	Counting and sampling surviving coral larvae and juveniles after their experimental exposure to heat stress was conducted using established methods in the field.<br>Sample sizes were chosen for larvae to maximize the number of larvae per well for DNA and RNA extraction (n = 30) but still allowed for accurate counting (optimum is 10).<br>Sample size were chosen for juveniles to optimize the number of juveniles that could be induced to metamorphose whilst maximizing the probability of at least n = 5 individuals surviving by the end of the experiment.
Data collection	Physiological data on survival was collected at the larval and juvenile stage.<br>Larval counts were conducted by having one person with a light standing over the wells and counting the number of individuals swimming, whilst a second person recorded the data. Juveniles were photographed with a Nikon D810 with a Nikon AF-S 60 mm f/2.8 G Micro ED Lens with four Ikelite DS160 Strobes. Photographs were analyzed in ImageJ.
Timing and spatial scale	Larval survival was counted from 0 to 56 hours at 27°C and 35.5°C.<br>Juvenile survival was counted at 0 and after 58 days at 27 and 32°C treatments.<br>Larvae were assayed for RNAseq at 0 and 56 hours, and juveniles only at 58 days.<br>The spatial scale for which these colonies were collected were from latitudes -12.50029 (degS) to -18.821744 (degS). See manuscript for map of locations. Experiments were carried out from Nov 2018 until March 2019.<br>Counts and sampling was performed when the average survival across all crosses in the heat treatment reached approximately 50%.
Data exclusions	No data were excluded. All data are found via Github.
Reproducibility	Replicate biological crosses were made within the experiment. These are biological replicates that are included as replicates within the statistical design. This experiment has not been repeated to verify reproducibility.
Randomization	Randomization of individual larvae and juveniles was carried across across all replicate wells, plates, tanks, and temperatures.
Blinding	Given the randomization of familial crosses within the larval and juvenile experiments, it was impossible to know which family was being counted (therefore blind to cross identity). However, we could not be blind to tank temperature as when you are counting you can feel the difference in temperature between ambient and warm treatments. Symbiont identity was also not blinded for the juvenile experiment as the phenotypes of juveniles between the four treatments was very different.

Did the study involve field work? ☒ Yes ☐ No

Field work, collection and transport

Field conditions	The relevant study sites (location of coral collections) and their temperature ranges are shown in Supplementary Figure 1. Gravid Acropora tenuis colonies were sourced from three (CU-Curd, LS-Long Sandy, SB-Sand Bank 7) and two reefs (BK-Backnumbers, DR-Davies) in the far northern and central GBR, respectively, encompassing a 23 - 33°C of the 1.5x interquartile range of the mean annual temperature gradient (>6° of latitude, ~900 km). Curd on average is the warmest reef.
Location	Great Barrier Reef (Australia). The field parameters of each site are described extensively in Supplementary Figure 1. These included, in the north, Curd (CU: −12.5850◦S, 143.5115◦E), Sand Bank 7 (SB: −13.4362◦S, 143.9714◦E), Long Sandy (LS: −12.5003◦S, 143.7848◦E), Davies (DR: −18.8217◦S, 147.6495◦E), and Backnumbers (BK: −18.5075◦S, 147.1464◦E) reefs in the central region. Water depth of collections ~4m.
Access & import/export	All field work and collection and care of laboratory animals were conducted under institutional guidelines and permit numbers G12/35236.1 and G18/41667.1.
Disturbance	Colonies were collected from the reef without disturbing the surrounding benthos as per permit conditions.

Reporting for specific materials, systems and methods

We require information from authors about some types of materials, experimental systems and methods used in many studies. Here, indicate whether each material, system or method listed is relevant to your study. If you are not sure if a list item applies to your research, read the appropriate section before selecting a response.

Materials & experimental systems

n/a	Involved in the study
☒	☐ Antibodies
☒	☐ Eukaryotic cell lines
☒	☐ Palaeontology and archaeology
☐	☒ Animals and other organisms
☒	☐ Clinical data
☒	☐ Dual use research of concern

Methods

n/a	Involved in the study
☒	☐ ChIP-seq
☒	☐ Flow cytometry
☒	☐ MRI-based neuroimaging

Animals and other research organisms

Policy information about studies involving animals; ARRIVE guidelines recommended for reporting animal research, and Sex and Gender in Research

Laboratory animals	Laboratory animals were produced by reproductively crossing field-collected, wild animals. All animals were from Acropora tenuis.
Wild animals	Wild caught reproductive adults of Acropora tenuis were collected from the field. Each likely around 3 to 5 years of age. Individual corals were collected using chisel and hammer from the reef by divers on SCUBA. Animals were transported via plane or boat to the aquarium facility and house in separate tanks set to the daily temperature variation cycle of their reef of origin. Adults were killed during a mass-flooding, extreme-weather event at the facility after these experiments.
Reporting on sex	Study organism is hermaphroditic.
Field-collected samples	Wild caught, field-collected reproductive adults of Acropora tenuis were collected from the field. Each likely around 3 to 5 years of age. Individual corals were collected using chisel and hammer from the reef by divers on SCUBA. Animals were transported via plane or boat to the aquarium facility and house in separate tanks set to the daily temperature variation cycle of their reef of origin. Adults were killed during a mass-flooding, extreme-weather event at the facility after these experiments.
Ethics oversight	Corals are not subject to Ethics oversight within Australia. All field work and collection and care of laboratory animals were conducted under institutional guidelines and permit numbers G12/35236.1 and G18/41667.1

Note that full information on the approval of the study protocol must also be provided in the manuscript.
